# RON is not a prognostic marker for resectable pancreatic cancer

**DOI:** 10.1186/1471-2407-12-395

**Published:** 2012-09-07

**Authors:** Carole M Tactacan, David K Chang, Mark J Cowley, Emily S Humphrey, Jianmin Wu, Anthony J Gill, Angela Chou, Katia Nones, Sean M Grimmond, Robert L Sutherland, Andrew V Biankin, Roger J Daly

**Affiliations:** 1 Cancer Research Program, Garvan Institute of Medical Research, 384 Victoria St, Darlinghurst, Sydney, NSW 2010, Australia; 2 Department of Surgery, Bankstown Hospital, Eldridge Road, Bankstown, Sydney, NSW 2200, Australia; 3 South Western Sydney Clinical School, Faculty of Medicine, University of NSW, Liverpool, NSW 2170, Australia; 4 Department of Anatomical Pathology, Royal North Shore Hospital, St Leonards, Sydney, NSW 2065, Australia; 5 Northern Clinical School, Faculty of Medicine, University of Sydney, Sydney, NSW 2006, Australia; 6 Department of Anatomical Pathology, St. Vincent’s Hospital, Darlinghurst, Sydney, NSW 2010, Australia; 7 Queensland Centre for Medical Genomics, Institute for Molecular Bioscience, The University of Queensland, Brisbane, QLD 4072, Australia; 8 Australian Pancreatic Cancer Genome Initiative Consortium, The Kinghorn Cancer Centre, Garvan Institute of Medical Research, 372 Victoria Street, Darlinghurst, Sydney, NSW 2010, Australia

**Keywords:** Receptor tyrosine kinase, Biomarker, Gemcitabine, Chemotherapy

## Abstract

**Background:**

The receptor tyrosine kinase RON exhibits increased expression during pancreatic cancer progression and promotes migration, invasion and gemcitabine resistance of pancreatic cancer cells in experimental models. However, the prognostic significance of RON expression in pancreatic cancer is unknown.

**Methods:**

RON expression was characterized in several large cohorts, including a prospective study, totaling 492 pancreatic cancer patients and relationships with patient outcome and clinico-pathologic variables were assessed.

**Results:**

RON expression was associated with outcome in a training set, but this was not recapitulated in the validation set, nor was there any association with therapeutic responsiveness in the validation set or the prospective study.

**Conclusions:**

Although RON is implicated in pancreatic cancer progression in experimental models, and may constitute a therapeutic target, RON expression is not associated with prognosis or therapeutic responsiveness in resected pancreatic cancer.

## Background

Pancreatic cancer (PC) still remains one of the most aggressive and lethal of human cancers, with a lack of prognostic biomarkers and effective treatments contributing to its poor prognosis and high mortality. Around 15% of PC patients are eligible to undergo potentially curative pancreatic surgery at the time of presentation. However, those with operable PC still only have an 18–23% 5-year survival [1,2], with high incidences of local recurrence and hepatic metastases occurring within 1–2 years after surgery [3]. This emphasizes the need for adjuvant intervention. Gemcitabine is the current standard for post-operative chemotherapy and delays the development of recurrent disease in some PC patients [4]. One explanation for why only a subset of PC patients respond favourably to adjuvant treatment is the molecular heterogeneity of PC [5]. The concept of stratifying patients based on their molecular signatures has been successful in breast cancer and is the basis of modern clinical oncology [6]. Implementation of this treatment strategy for PC may also prove beneficial. The literature is replete with biomarkers of prognosis and therapeutic responsiveness identified through small and/or single pancreatic cancer patient cohorts. However, a recent systematic review has shown that very few of these have been independently validated [7]. The calcium binding protein S100A2 is one of the few prognostic biomarkers where this is the case [7]. In PC, tumors that are negative for S100A2 have a significant survival benefit with pancreatectomy compared to tumors with moderately-high to high expression [8].

Recepteur d’origine nantais (RON), also referred to as macrophage stimulating 1 receptor (MST1R), is a receptor tyrosine kinase (RTK) and member of the mesenchymal epithelial transition factor (MET)-proto-oncogene family. Similar to other cell surface receptors, RON is activated upon binding of its ligand, the hepatocyte growth factor-like (HGFL) protein, also referred to as macrophage stimulating protein (MSP) and macrophage stimulating 1 (MST1). HGFL is synthesized from hepatocytes and secreted as an endocrine mediator in an inactive precursor form called pro-MSP. Subsequently, the type II transmembrane proteinase MT-SP1, also known as suppression of tumorigenicity 14 (ST14), processes pro-MSP to its active form near the cell surface, where it activates RON, leading to the initiation of multiple signaling cascades that impact upon cellular motility and survival [9].

RON is overexpressed in PC relative to the ductal epithelium of non-malignant pancreas, and its signaling enhances migration, invasion, and survival of PC cells and promotes resistance to gemcitabine in experimental models, thus making it a potential therapeutic target and a possible marker of prognosis [10-12]. Although RON is associated with poor survival in gastroesophageal cancer [13] and a three-gene signature involving RON, MSP, MT-SP1 is a strong indicator for metastasis and poor prognosis in breast cancer [14], the prognostic significance of RON in PC remains unknown. The goal of this study was to use a comprehensive cohort of PC patients to assess RON as a biomarker of prognosis and therapeutic responsiveness.

## Methods

### Optimization of the RON antibody for immunohistochemistry

Anti-RON β (C-20) antibody (Santa Cruz Biotechnology) was used to immunoblot for RON expression across a panel of PC cell lines; this antibody detects full-length pro-RON (190 kDa), mature RON β-chain (150 kDa), and a short form sf-RON isoform (55 kDa) [15]. MIA PaCa-2 and Panc 10.05 were selected as low-expressing and high-expressing controls for RON expression, respectively, and were processed as formalin-fixed, paraffin-embedded blocks and subsequently sectioned at 4 μm onto positively charged slides (Superfrost plus; Menzel-Glaser, Braunschweig, Germany). Antigen was retrieved using DAKO S1699 solution in a pressure cooker for 1 min. Immunostaining was performed using a DAKO Auto-stainer. The slides were treated with 3% Peroxidase Block (DAKO, K4011) for 5 min then Protein Block (DAKO, X0909) for 10 min and stained with primary anti-RON β (C-20) antibody at a dilution of 1:100 for 60 min. EnVision + System anti-rabbit (DAKO, K4003) was used as secondary antibody then 3,3'-diaminobenzidine (DAKO, K3468) was used as a substrate. The slides were then counterstained with Mayer’s haematoxyline.

### Patients, tissue microarrays, immunohistochemistry and statistical analysis

Clinico-pathologic and outcome data for 492 consecutive patients with a diagnosis of pancreatic ductal adenocarcinoma who underwent pancreatic resection were obtained from teaching hospitals associated with the Australian Pancreatic Cancer Network [16] (Table 1). This cohort consisted of a training set of 76 patients, a validation set of 316 patients and a further cohort of 100 patients accrued prospectively for the International Cancer Genome Consortium (ICGC) [17]. Detailed methods for tissue microarray construction, the assessment of immunostaining and statistical analysis were described previously [8].

**Table 1:** Clinico-pathological and outcome data corresponding to the pancreatic cancer tissue microarray training and validation sets, and the prospective ICGC cohort gene expression array

	**Training Cohort**			**Validation Cohort**			**ICGC Cohort**		
**Variables**	**n = 76 No. (%)**	**Median DSS (months)**	***P*value (Logrank)**	**n = 316 No. (%)**	**Median DSS (months)**	***P*value (Logrank)**	**n = 100 No. (%)**	**Median DSS (months)**	***P*value (Logrank)**
**Sex**									
Male	45 (59.2)	16.3		157 (49.7)	18.3		61 (61.0)	18.4	
Female	31 (40.8)	8.5	0.0340	159 (50.3)	16.9	0.5792	39 (39.0)	18.3	0.5467
**Age (years)**									
Mean	62.1			66.7			66.9		
Median	64.5			69.0			68.0		
Range	35.0 – 83.0			28.0 – 87.0			34.0 – 90.0		
**Outcome**									
Follow-up (months)	0.3 – 158.0			0.1 – 195.8			0.1 – 29.8		
Median follow-up	158.0			68.7			14.1		
Death PC	68 (89.5)			259 (82.0)			33 (33.0)		
Death other	5 (6.6)			15 (4.7)			9 (9.0)		
Death Unknown	0 (0.0)			3 (0.9)			3 (3.0)		
Alive	1 (1.3)			38 (12.0)			55 (55.0)		
Lost to FU	2 (2.6)			1 (0.3)			0 (0.0)		
**Stage**^**a**^									
I	16 (21.1)	19.6		23 (7.3)	41.0		8 (8.0)	17.4	
II	59 (77.6)	11.5		282 (89.2)	17.8		87 (87.0)	18.8	
III	0 (0.0)			0 (0.0)			1 (1.0)	----^b^	
IV	1 (1.3)	22.0	0.2828	11 (3.5)	7.6	<0.0001	4 (4.0)	12.0	****^c^
**T Stage**									
T1	12 (15.8)			16 (5.1)			3 (3.0)		
T2	29 (38.2)	13.6		33 (10.4)	26.6		12 (12.0)	17.4	
T3	35 (46.1)	14.7	0.4857	267 (84.5)	16.8	0.0084	84 (84.0)		
T4	0 (0.0)			0 (0.0)			1 (1.0)	18.4	0.4297
**N Stage**									
N0	37 (48.7)	19.8		119 (37.7)	21.2		24 (24.2)	17.4	
N1	39 (51.3)	9.7	<0.0001	197 (62.3)	16.7	0.0267	75 (75.8)	18.4	0.4714
**Grade**									
I	7 (9.2)			26 (8.2)			4 (4.0)		
II	43 (56.6)	15.0		209 (66.1)	17.7		61 (61.0)	----	
III	26 (34.2)	11.2	0.0283	81 (25.6)	18.3	0.5971	33 (33.0)		
IV							2 (2.0)	15.1	0.0011
**Tumor size**									
≤ 20 mm	15 (19.7)	17.1		77 (24.6)	32.0		14 (14.0)	18.3	
> 20 mm	61 (80.3)	11.9	0.1232	236 (75.4)	16.0	<0.0001	86 (86.0)	18.4	0.8056
**Margins**									
Clear	40 (52.6)	18.6		195 (61.7)	22.4		66 (66.0)	----	
Involved	36 (47.4)	9.7	0.0004	121 (38.3)	13.3	<0.0001	34 (34.0)	13.9	0.0335
**Tumor Location**									
Head	62 (81.6)	15.6		258 (81.6)	18.8		85 (85.0)	18.4	
Others	14 (18.4)	7.4	0.0004	58 (18.4)	13.0	0.0312	15 (15.0)	13.6	0.0488
**Perineural Invasion**									
Negative	24 (32.0)	15.6		82 (26.6)	25.6		20 (20.6)	----	
Positive	51 (68.0)	13.6	0.1909	226 (73.4)	17.4	0.1180	77 (79.4)	17.4	0.0211
**Vascular Invasion**									
Negative	45 (60.0)	15.0		161 (53.5)	21.2		39 (40.6)	----	
Positive	30 (40.0)	10.1	0.0141	140 (46.5)	16.2	0.0070	57 (59.4)	15.9	0.0348
**Adj Chemotherapy**									
Yes	13 (17.1)	13.6		98 (31.0)	22.4		65 (68.4)	21.4	
No	63 (82.9)	14.1	0.7737	218 (69.0)	16.5	0.0451	30 (31.6)	12.0	0.0007
**RON Expression**^**d**^									
Low or absent	51 (79.7)	15.0		265 (94.3)	17.1		42 (52.5)	9.8	
High	13 (20.3)	6.4	0.0409	16 (5.7)	18.3	0.2799	38 (47.5)	8.0	0.3830

Patient Cohorts Characteristics.

^a^ AJCC 7^th^ Edition.

^b^ Median survival has not been reached yet.

^c^ Rank test cannot be tested as one or more groups contained no censored observations.

^d^ High RON expression was defined as H > 210 for immunohistochemistry or greater than 8.74 on normalized Log2 score for gene expression array.

Immunostaining was performed on the training and validation set using the anti-RON β (C-20) antibody as described above. Positive RON expression was defined as a modified histoscore (intensity x %) >210 as this was the most discriminant cut-off point in the training set. Median survival was estimated using the Kaplan-Meier method and the difference was tested using the log-rank Test. *P* values of less than 0.05 were considered statistically significant. Statistical analysis was performed using StatView 5.0 Software (Abacus Systems, Berkeley, CA, USA). Disease-specific survival was used as the primary endpoint.

### Expression array analysis

The pancreatic ICGC cohort consists of prospectively acquired, primary operable, non-pretreated pancreatic ductal adenocarcinoma samples [18]. For the ICGC cohort, tumour cells were enriched by macro-dissection, then RNA was extracted from tumors using Qiagen Allprep® (Qiagen, Valencia, CA) in accordance with the manufacturer's instructions, assayed for quality on an Agilent Bioanalyzer 2100 (Agilent Technologies, Palo Alto, CA), and hybridized to Illumina Human HT-12 V4 microarrays. mRNA expression data were available for 88 of 100 patients. Raw iDAT files were processed using *IlluminaGeneExpressionIdatReader* (Cowley *et al.* manuscript in preparation). Following array quality control, data were vst transformed and robust spline normalized, using the *lumi* R/Bioconductor package [19]. To confirm the microarray probe quality, we: aligned the probe sequences to the genome using UCSC BLAT [20]; and also used an Illumina reannotation pipeline [21]. Both methods confirmed that the probes for RON, MSP, MT-SP1 perfectly and uniquely match the 3’ end of the intended gene. The RON probe binds both full length RON, and sf-RON. Expression levels of single-gene (RON, MSP, MT-SP1), two-gene (RON + MSP) and three-gene (RON + MSP + MT-SP1) combinations were used to separate patients into two groups: high for those patients with above-mean expression in all genes in the signature, or low for all other patients. For RON, we also chose an 80% low : 20% high cutoff to match that proportion of RON high patients in the training cohort. Survival analysis was performed using the Cox proportional hazards model, using the *survival* package (version 2.36-9) in R (version 2.13.1). Expression levels for all signatures were also analyzed in a subset of 65 patients from the ICGC cohort, which omitted patients with advanced disease.

## Results

### Optimization of anti-RON antibody for immunohistochemistry

**Figure 1:**
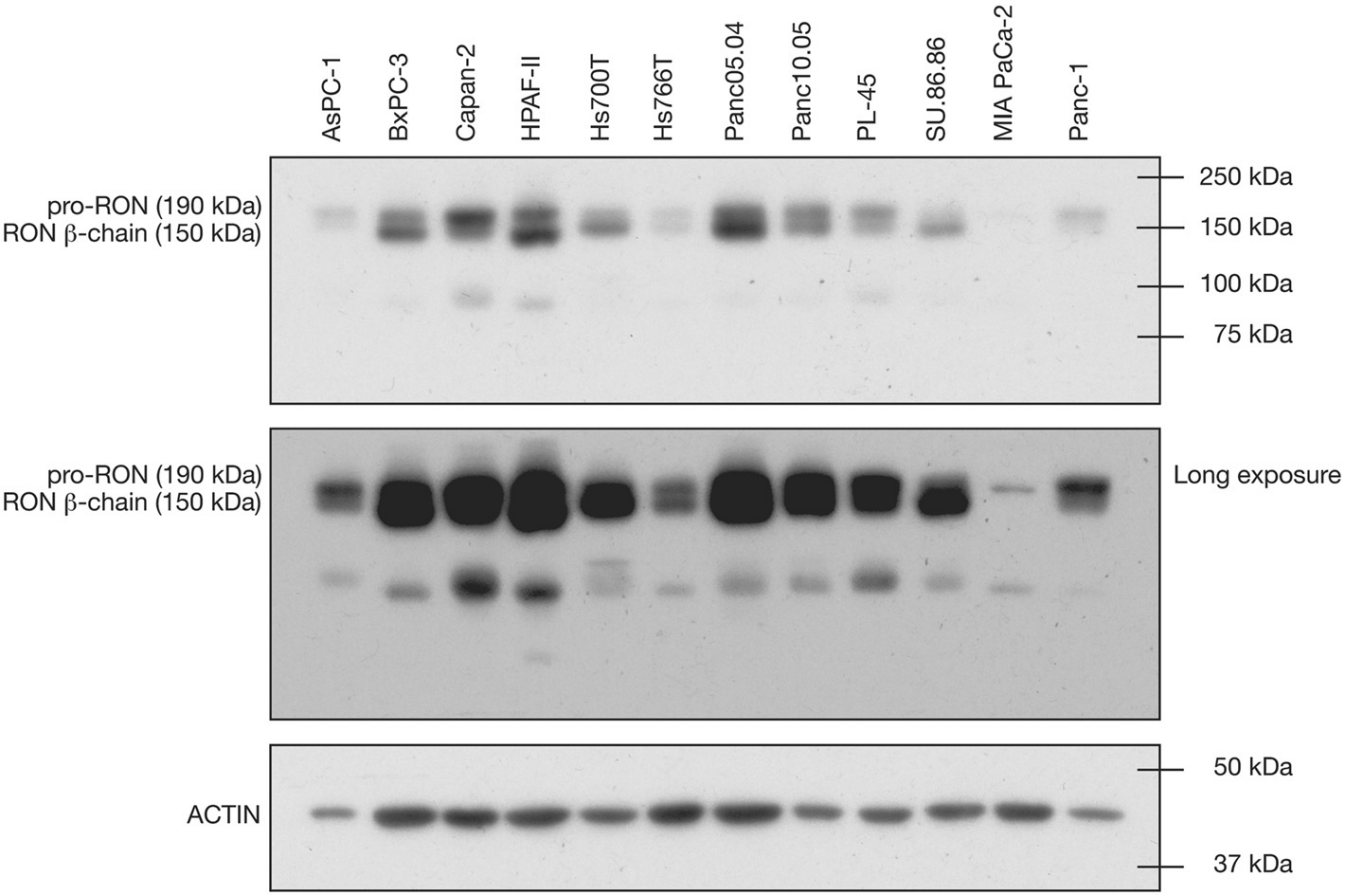
**Western blot of pro-RON and RON β-chain expression across a panel of pancreatic cancer cell lines.** MIA PaCa-2 and Panc 10.05 are low-expressing and high-expressing controls, respectively.

**Figure 2:**
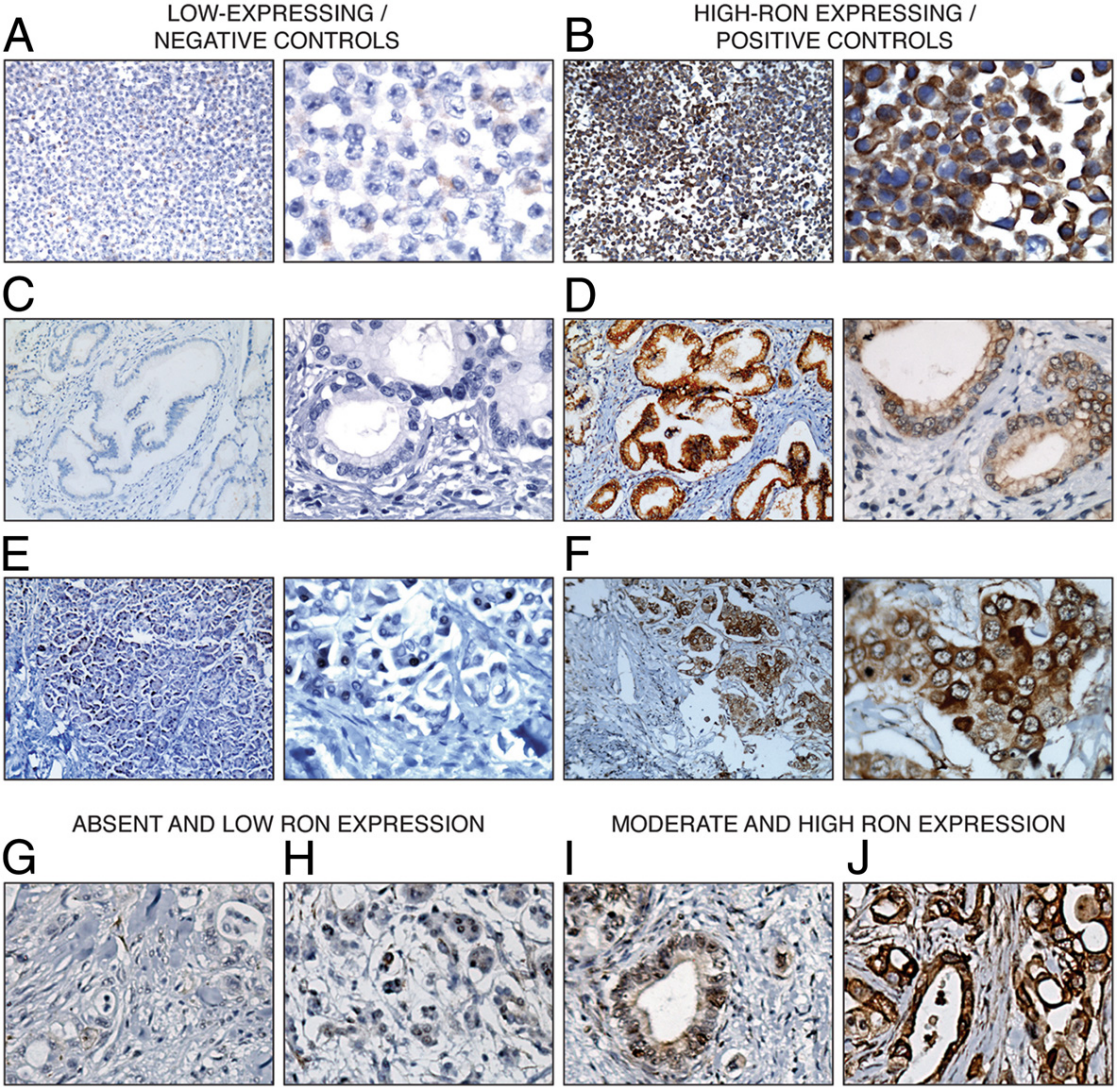
**Optimization of RON immunohistochemical staining.** Immunohistochemical staining of RON in MIA PaCa-2 and Panc 10.05 cell lines (**A** and **B**), using IgG rabbit and anti-RON primary antibodies in pancreatic cancer tissues (**C** and **D**), and in benign ductal cells of the pancreas and in breast cancer with high RON expression (**E** and **F**), at low and high magnification. Examples of absent, low, moderate to high RON staining in pancreatic cancer tissue are also shown (**G**-**J**).

RON protein expression was determined by Western blot analysis across a panel of PC cell lines (Figure 1). All cell lines expressed RON, but at varying levels. These results were used to select appropriate low-expressing and high-expressing control cell line blocks for subsequent immunohistochemistry. MIA PaCa-2 was used as the low-expressing control (Figure 2A), and Panc 10.05 was used as the high-expressing control (Figure 2B). The anti-RON antibody gave strong staining of a subset of pancreatic cancers (Figure 2D, I & J) that was absent upon use of control rabbit IgG (Figure 2C). Examples where RON expression in pancreatic cancer was undetectable, or low, are also shown (Figure 2G and H). RON is rarely expressed in the ductal cells of non-cancerous pancreas [3], so this was used as the negative-control tissue (Figure 2E). RON is known to be overexpressed in breast cancer [14], which provided an additional positive-control tissue (Figure 2F). Staining was present in both membrane and cytoplasm, which is consistent with past literature [10,12].

### RON expression in pancreatic cancer and its association with patient prognosis

**Figure 3:**
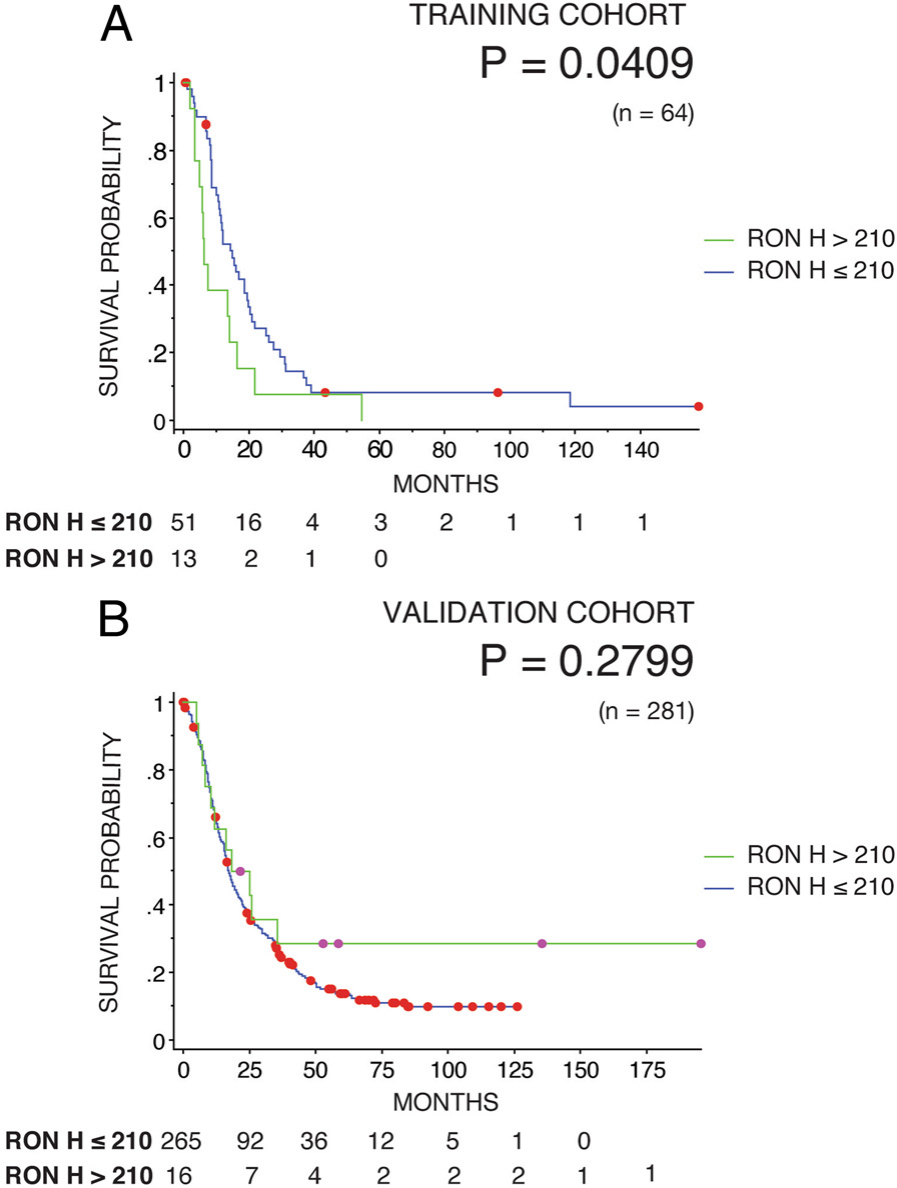
**Kaplan-Meier survival curves for patients stratified based upon RON expression in the training (n = 64, with n = 51 low and n = 13 high) (A) and validation (n = 281, with n = 265 low and n = 16 high) (B) cohorts.** The p-value from fitting a Cox proportional hazards model to the survival curves is indicated in the plots.

**Figure 4:**
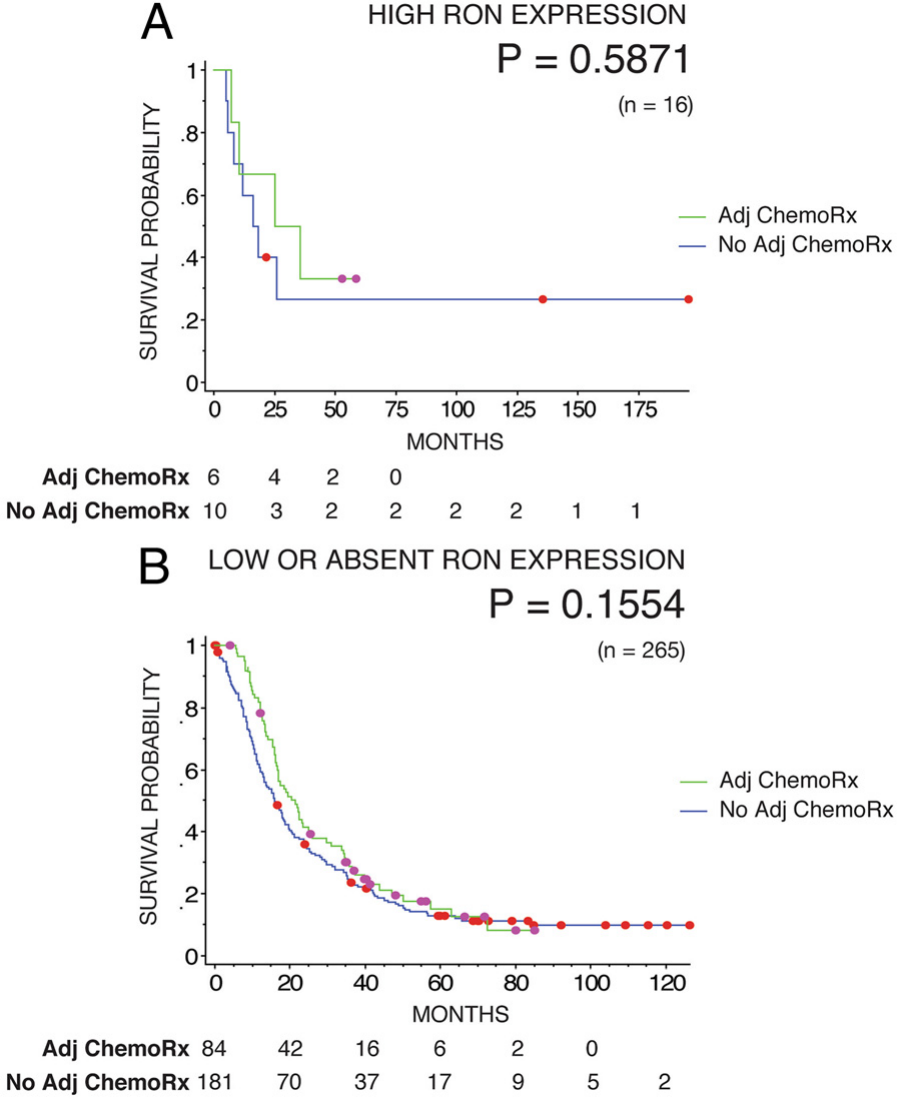
**Kaplan-Meier survival curves for patients stratified according to adjuvant chemotherapy status with high RON expression (n = 16) (A) and low or absent RON expression (n = 265) (B) in the validation cohort (n = 281).** The p-value from fitting a Cox proportional hazards model to the survival curves is indicated in the plots.

Three independent patient cohorts were utilized for this study. Two of the cohorts, the training and validation sets, which were acquired retrospectively, were immunohistochemically stained for RON expression. High RON expression (H-score >210) was a biomarker of poor prognosis in the training set (Figure 3A). However, in the larger validation set RON expression was not prognostic (Figure 3B). The evaluation of RON as a predictive marker in the response to adjuvant gemcitabine therapy was not investigated in the training set as this cohort pre-dates adjuvant gemcitabine given as standard treatment. In the validation set, RON expression did not co-segregate with chemotherapy responsiveness, however a trend towards better qualitative response was seen for the RON low or absent group (Figure 4A & B).

Additionally, there was no association between RON expression and tumor stage (Chi-squared *P* = 0.123), tumor size (*P* = 0.629) lymph node metastases (*P* = 0.942), grade (*P* = 0.332), perineural (*P* = 0.335) or vascular invasion (*P* = 0.210).

**Figure 5:**
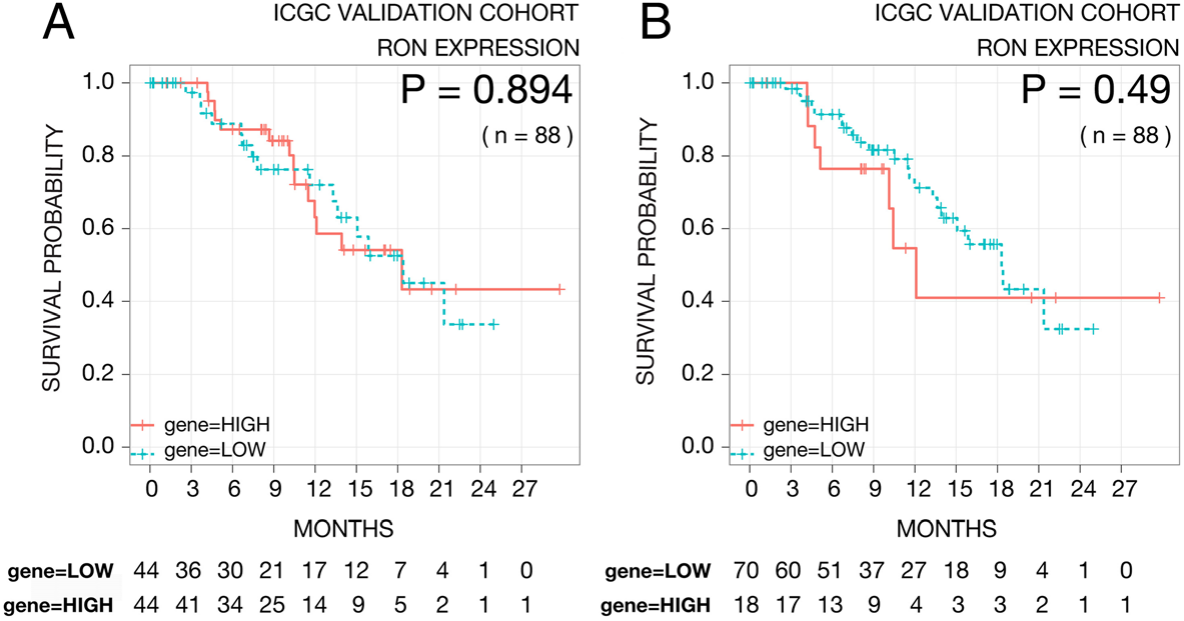
**Kaplan-Meier survival curves for patients stratified based upon RON mRNA expression in the prospectively accrued ICGC cohort of PC (n = 88).** Patients were split into two groups using a 50% low : 50% high cutoff (n = 44 in both groups) (**A**), or an 80% low : 20% high cutoff (n = 70 and n = 18, respectively) which matches the cutoff found using protein expression in the training cohort (**B**). The p-value from fitting a Cox proportional hazards model to the survival curves is indicated in the plots.

**Figure 6:**
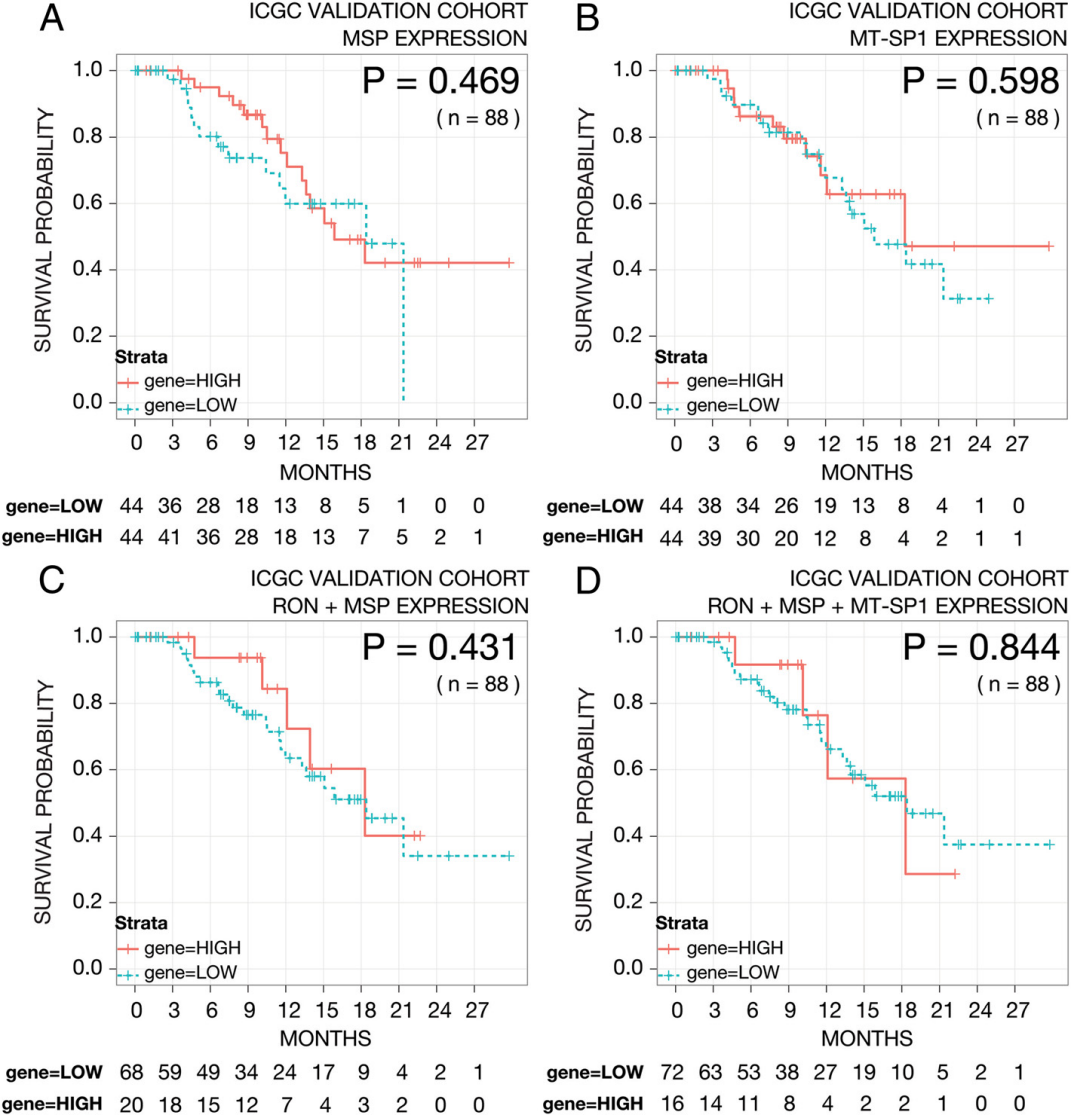
**Kaplan-Meier survival curves for patients stratified based upon mRNA expression levels of MSP (A), MT-SP1 (B), a two-gene signature combination of RON + MSP expression (C), and a three-gene signature combination of RON + MSP + MT-SP1 expression (D) from the prospectively accrued ICGC cohort (n = 88).** All genes use a 50%:50% low:high cutoff. The p-value from fitting a Cox proportional hazards model to the survival curves is indicated in the plots.

RON mRNA was expressed in a continuous manner over a third, prospectively acquired cohort, so two cutoffs were used: a 50%:50% (Figure 5A), and an 80%:20% (Figure 5B) low:high cutoff to match the frequency utilized in the retrospective cohorts subjected to immunohistochemistry. At neither cutoff was RON associated with prognosis. We omitted a group of patients with more advanced disease (leaving n = 65), and again, RON was not associated with prognosis (data not shown). In addition, MSP or MT-SP1 expression (Figure 6A & B), a two-gene expression signature of RON + MSP (Figure 6C), and a three-gene expression signature of RON + MSP + MT-SP1 (Figure 6D), were also investigated, however, none of these signatures were associated with prognosis.

## Discussion

Pre-clinical studies identified RON as a potential predictive marker for gemcitabine response in pancreatic cancer [11,12]. This study examined RON as a prognostic and predictive biomarker in three large well-annotated cohorts of patients (total of 492) with resectable PC using immunohistochemistry for the training and validation set and gene expression arrays for the ICGC cohort. Apart from the training cohort, RON expression was not associated with survival. The validation cohort consisted of 316 patients and had a 99% power to detect a hazard ratio of 1.90 (assuming it has a HR and an expression pattern similar to the training cohort with a Type I error of 0.05). However, RON overexpression was only observed in 5.4% of patients in the validation set as compared to 22.1% in the training set, but the reason for the much lower proportion of RON over-expressers in the validation set is not known. The unexpected lower proportion of the over-expressers in the validation cohort has reduced the power to detect a difference and therefore, the inability to detect a difference may be due to a Type 2 error rather than there being truly no difference between the two groups. A post-hoc analysis of the validation set using different cut-off points for RON expression could not demonstrate any differential survival (data not shown). It is therefore likely that due to the smaller number of patients, the training cohort returned a false-positive result, which highlights the importance of independent validation in biomarker discovery and development.

Both the antibody used for IHC and the probe used for mRNA analysis recognized the same RON isoforms: the full-length inactive precursor pro-RON, the active RON β-chain and short form sf-RON [15], thus enabling comparisons to be made between the cohorts.

RON mRNA was expressed in a continuous manner in the third, prospectively acquired cohort, so two cutoffs were used: the mean (Figure 5A), and an 80%:20% low:high cutoff to match the frequency utilized in the restrospective cohorts (Figure 5B). Using both approaches, RON was not associated with prognosis. Additional to RON expression alone, a combination gene signature of RON, MSP and MT-SP1, which is prognostic for breast cancer, was also evaluated. However, in PC this three-gene signature, and even two-gene signature combinations, did not correlate with patient survival. We acknowledge that there is reduced power in the 2-gene and 3-gene signatures due to low sample numbers in each strata, but it should be noted that this is the largest prospectively accrued cohort of PC patients with mRNA data to date. Collectively, this suggests that the role of RON is cancer-type specific and that RON is unlikely to be a major metastatic driver in PC. However, the ICGC cohort is still relatively young, currently with a median follow up of 14.1 months, and exhibits relatively small numbers. Therefore, the negative result can also possibly be due to the study being under-powered.

Data from this study do not support RON as a prognostic or predictive biomarker in resectable PC. This study, however, did demonstrate that RON is expressed in a large proportion of PC (training set: 61 of 64, 95.3%; validation set: 253 of 281, 90.0%), which is consistent with previous published data [10]. Due to increased expression during PC progression RON may still prove to be an effective therapeutic target.

To improve the efficiency of gemcitabine in PC, several studies have evaluated the co-administration of gemcitabine with other cytotoxic and biological agents [22,23]. However, the epidermal growth factor receptor (EGFR) inhibitor, erlotinib, was the only agent to show efficacy in a phase III setting [24]. The EGFR is commonly overexpressed in PC and is associated with poor prognosis and disease progression [25]. The frequent overexpression of RON in PC suggests that it could also be a target for therapy. Supporting this concept, silencing RON expression reduced growth of pancreatic cancer xenografts and increased gemcitabine-induced apoptosis [11]. In addition, function-blocking monoclonal antibodies and small molecular inhibitors directed against RON have demonstrated promising results in pre-clinical models [10,26,27].

## Conclusion

RON is not associated with prognosis or therapeutic responsiveness in resectable PC, in the current cohorts of patients. However it may still represent a therapeutic target in this disease.

## Abbreviations

EGFR: Epidermal growth factor receptor; HGFL: Hepatocyte growth factor-like; H-score: Histoscore; HR: Hazard ratio; ICGC: International Cancer Genome Consortium; MET: Mesenchymal epithelial transition factor; MSP: Macrophage stimulating protein; MT-SP1: Membrane-type serine protease 1; PC: Pancreatic cancer; RON: Recepteur d’origine nantais; RTK: Receptor tyrosine kinase.

## Competing interests

The authors declare that they have no competing interests.

## Authors’ contributions

RJD and AVB devised, planned and co-ordinated the study. ESH identified RON to have variable expression levels in pancreatic cancer. CMT carried out the immunohistochemical staining of RON in the training and validation set of pancreatic cancer tissue microarray patient cohorts. AJG and AC independently scored the tissue microarray cohorts, providing a histoscore for quantification. DKC analyzed the histoscores with the accompanying clinico-pathological and patient outcome data. SMG and KN are associated with the ICGC. KN carried out the gene expression profiling in the ICGC cohort. MJC and JW carried out the analysis of the ICGC cohort. CMT and DKC drafted the manuscript. All authors read, revised and approved the final manuscript.

## Pre-publication history

The pre-publication history for this paper can be accessed here:

http://www.biomedcentral.com/1471-2407/12/395/prepub
